# A framework to support the progressive implementation of integrated team-based care for the management of COPD: a collective case study

**DOI:** 10.1186/s12913-022-07785-x

**Published:** 2022-03-30

**Authors:** Shannon L Sibbald, Vaidehi Misra, Madelyn daSilva, Christopher Licskai

**Affiliations:** 1 Faculty of Health Sciences, University of Western Ontario, 1151 Richmond St, HSB-334, London, ON N6A 2K5 Canada; 2 Department of Family Medicine, Schulich School of Medicine and Dentistry, University of Western Ontario, 1151 Richmond St, HSB-334, London, ON N6A 2K5 Canada; 3 Department of Medicine, Schulich School of Medicine and Dentistry, University of Western Ontario, London, Canada

**Keywords:** Implementation science, Primary care, Evidence based practice, Patient care team, Integrated team-based care, Chronic obstructive pulmonary disease

## Abstract

**Background:**

In Canada, there is widespread agreement about the need for integrated models of team-based care. However, there is less agreement on how to support the scale-up and spread of successful models, and there is limited empirical evidence to support this process in chronic disease management. We studied the supporting and mitigating factors required to successfully implement and scale-up an integrated model of team-based care in primary care.

**Methods:**

We conducted a collective case study using multiple methods of data collection including interviews, document analysis, living documents, and a focus group. Our study explored a team-based model of care for chronic obstructive pulmonary disease (COPD) known as Best Care COPD (BCC) that has been implemented in primary care settings across Southwestern Ontario. BCC is a quality improvement initiative that was developed to enhance the quality of care for patients with COPD. Participants included healthcare providers involved in the delivery of the BCC program.

**Results:**

We identified several mechanisms influencing the scale-up and spread of BCC and categorized them as Foundational (e.g., evidence-based program, readiness to implement, peer-led implementation team), Transformative (adaptive process, empowerment and collaboration, embedded evaluation), and Enabling Mechanisms (provider training, administrative support, role clarity, patient outcomes). Based on these results, we developed a framework to inform the progressive implementation of integrated, team-based care for chronic disease management. Our framework builds off our empirical work and is framed by local contextual factors.

**Conclusions:**

This study explores the implementation and spread of integrated team-based care in a primary care setting. Despite the study’s focus on COPD, we believe the findings can be applied in other chronic disease contexts. We provide a framework to support the progressive implementation of integrated team-based care for chronic disease management.

## Background

Integrated team-based models of care have emerged as a means to improve care delivery and promote system sustainability [1]. Canadian provinces continue to implement integrated models of care; for example, Canada’s most populous province, Ontario, is currently undergoing significant restructuring to better integrate its healthcare system [2]; interprofessional and integrated team-based care are at the center of the reform. In the past, much of implementation occurred with a short-term focus on local implementation with limited attention to spread, scale-up, or sustainability [3]. Indeed, there is a lack of guidance in the literature on how to account for, and support, contextual differences while maintaining the fidelity of successful models. It is unclear whether and how these models will work efficiently in different contexts [4].

The shift towards integrated team-based care can be observed in the management of chronic obstructive pulmonary disease (COPD) [5]. Globally, COPD is a leading cause of morbidity, mortality, and health resource consumption [6]. The burden of COPD is compounded by comorbidities (such as cardiac disease, depression, and anxiety), which require unique care interventions tailored to patients’ needs [7]. The growing prevalence of COPD, and its substantial impact on patients’ quality of life, require collaboration and coordination across the health sector to effectively manage patient health and prevent hospitalizations [8, 9]. The Best Care COPD (BCC) program delivers care within a primary care team setting and is built on collaboration between primary and specialist providers to deliver a care pathway tailored to patients’ needs [10]. Research on BCC has demonstrated the program’s ability to improve patient outcomes and reduce hospitalizations [11]. The success of the BCC program has led to its progressive implementation at several primary care sites across a geographic region.

Broadly, implementation efforts have been supported through several frameworks including the Consolidated Framework for Implementation Research (CFIR) [12], the Promoting Action of Research Implementation in Health Services (PARIHS) [13], and the Exploration, Preparation, Implementation, and Sustainment (EPIS) framework [14]. CFIR and PARIHS offer valuable insight to identify factors that can potentially have a central role in the implementation of health services [12, 13]. EPIS acknowledges the interplay of these factors through different phases of the implementation process and emphasizes the role of context [14]. While these frameworks have provided important insight, they have not been sufficiently applied to ‘progressive implementation’ [15] or spread and scale efforts. A recent publication conducted an overview of reviews in which they developed a conceptual framework that highlighted important constructs as the implementation process progresses; however, the authors acknowledged the lack of empirical findings as a limitation [16]. Most tools and frameworks do not use empirical research to account for the unique challenges of progressive implementation. We consider spread as progressive implementation - it refers to the horizontal expansion of a program to benefit more patients and/or providers [15]. Scale-up can be thought of as vertical implementation, occurring at the individual level (among patients, providers and staff), internal-setting level (e.g., leadership, resources, and infrastructure within the organization), and external-setting level (e.g., policy, resources, collaboration, and competition exhibited outside of the organization) [17].

We wanted to understand the progressive implementation of the BCC program across multiple primary care sites within the Southwestern region of Ontario, Canada. A previous phase of this research explored the initial spread of the program into one site [10]. This current study focuses on the second phase of implementation and includes an analysis across both phases. The progressive implementation of BCC to several primary care sites provided the opportunity to explore factors that impact the spread of integrated models of team-based care for patients with COPD, across diverse contexts.

## Methods

### Aim and design

We conducted a collective case study exploring the progressive implementation of BCC within one geographic region over multiple years (2019–2021) [18]. Each implementation site represents a single case in our collective case study approach; the phenomenon of interest across sites was the progressive implementation of the BCC program. Our research design and data collection tools were guided by the CFIR [10]; the Standards for Reporting Qualitative Research: a synthesis of recommendations (SRQR) was used for reporting accuracy [19].

### Sample and setting

Southwestern Ontario is home to nearly 1 million residents; approximately 30% of residents live in rural regions, 3% identify as Aboriginal, and 30% live below the provincial low-income cut-off [20]. Service delivery in this region is impacted by barriers to access including geography and a lack of after-hours care; these barriers are particularly prevalent when attempting to access primary care [21]. Southwestern Ontario exhibits a disparity in the distribution of comprehensive primary care physicians, with providers concentrated in densely populated areas and few physicians serving rural communities [22]. Further, team-based care is available to a minority primary care practices and where present, COPD specific programming is very uncommon. The BCC program aims to mitigate these barriers to access and provide comprehensive guideline-based care for patients.

BCC is a quality improvement initiative developed in 2009 by the Asthma Research Group Inc. (ARGI) to enhance the quality of care delivered to patients with COPD, within a primary care setting. One-on-one consultations with patients with COPD are conducted by a Respiratory Therapist (RT), RN, or other allied health provider. BCC providers hold an additional credential as a Certified Respiratory Educator (CRE). The program is designed to enable the educators to collaborate with the primary care site to proactively search to invite any patient at risk of COPD to the program. BCC providers work closely with the patient’s care team to develop an action plan, coordinate care, and educate patients about self-management.

The BCC program started in one geographic region of Southwestern Ontario, Canada and providers believed that it contributed to remarkable improvements in clinical outcomes, reduced emergency department visits, and improved patient quality of life [11, 23]. The program was implemented into a new primary care team (with five sites) in a neighbouring region as a proof-of-concept [10]. In 2018, the program was progressively implemented across a wider geography within Southwestern Ontario. At the time of our study, the program was comprised of nine educators (who were all RTs) across several sites (nine family health teams, two community health centres, and seven non-team based care clinics) with plans for continued growth within the region and across the province. Several sites were further divided into smaller clinics (or locations).

### Data collection processes

Multiple methods of data collection were used to develop an in-depth understanding of the progressive implementation of BCC. These included living documents (LDs), a focus group, interviews, and document analysis. The research team was an independent, objective party and possessed significant experience conducting semi-structured focus groups and interviews, and expertise in qualitative and mixed research methods. Participants were briefed on the purpose of the study and the data collection methodology in Consent Forms.

LDs are a semi-structured journaling approach [24] for gathering rich descriptions of participants’ experiences [25]; they provide key experiential knowledge of planned and unplanned implementation elements. Eight LDs with unique questions were distributed to each RT over a 10-week timeframe. Participants had, on average, 2 weeks to complete each LD within the 10-week time frame and received regular reminders.

A focus group was conducted with the nine providers responsible for implementing and delivering the BCC program. Questions were guided by CFIR, and informed by data collected in the LD to explore experiences of the implementation process and provider experience. CFIR provided a strong guiding framework for the focus group as it is a meta-theoretical framework incorporating a combination of constructs from several implementation frameworks [12]. This framework was able to provide a comprehensive perspective of the potential influences on implementation [12]. The findings from the LDs were used to further enhance the data collection tools to allow the research team to account for the dynamic and unique challenges of progressive implementation.

Interviews were conducted over the phone with resident primary care providers (physicians and nurse practitioners) from BCC implementation sites, who work collaboratively with the BCC CRE at their site, but that were external to BCC prior to implementation. Interviews explored implementation, provider experience, and impacts on care provision. The focus group and interviews involved the use of guides, spanned 1–2 h in length, and were audio recorded then transcribed for analysis.

Document analysis was used to advance the researchers’ knowledge of the BCC program’s implementation process through the contextual and background data. We collected existing team documents (such as meeting minutes, training documents, and memorandums of understanding) to develop a rich understanding of the context that supported our analysis.

### Data analysis

Data analysis was iterative and continuous; the research team relied on a conceptual and theoretical coding approach to identify themes [26]. Data was first analyzed independently by data source and then cross-analyzed. The first round of coding was done inductively (SLS and VM), looking for conversation, concepts, and ideas related to the implementation process. From this first round, key themes were pulled from the data and a coding framework was created. The second round of coding was conducted using our framework in a deductive approach (SLS, VM, and MD). Analysis was validated through triangulation and member checking [27, 28]. Participants and key informants were frequently consulted to discuss the accuracy and reliability of our findings; feedback was discussed when appropriate, and the findings were amended.

## Results

In total, there were 11 participants. All invited RTs participated in the LD and focus group (*n* = 9; response rate = 100%). One physician and one nurse practitioner participated in an interview (*n* = 2; response rate = 33%). The response rates for the living documents (*n* = 8) ranged from 44% to 89%. In total, we collected 47 documents. Our results are informed by all data sources across all sites and include verbatim quotes to demonstrate the themes that emerged through analysis.

Progressive implementation of BCC occurred in three phases: pre-implementation, implementation, and spread and sustainability (post-implementation). The phases built on one another and were mutually reinforcing. The success of each implementation phase was dependent on several mechanisms, which were categorized as foundational, transformative, and enabling (Table 1). Mechanisms acted as ‘input forces’ to move through implementation phases and reach the desired outcomes.

**Table 1:** Implementation phases and mechanisms

Category	Mechanism
Phases	
Implementation Phases	1. Pre-implementation 2. Implementation 3. Spread & Sustainability (post-implementation)
Mechanisms	
Foundational	1. Evidence-based Program 2. Readiness to Implement 3. Peer-led Implementation Team
Transformative	1. Adaptive Process 2. Empowerment and Collaboration 3. Embedded Evaluation
Enabling	1. Provider Training 2. Administrative Support 3. Role Clarity 4. Patient Outcomes

### Foundational mechanisms

Participants acknowledged their pre-implementation decision to implement the BCC program was multi-faceted. Three elements were foundational in pre-implementation: (1) an evidence-based program, (2) readiness to implement, and (3) implementation support. Each mechanism built on and supported the others.

Across sites, participants unanimously described BCC as being developed based on best practices and strong evidence. When creating the program, ARGI first identified existing programs and gaps within the care available to patients within their region. ARGI used this information to create evidence-based solutions to address patient and provider needs. Participants saw BCC as a multifaceted solution to manage care in a resource-strapped system.

“[this strategy was] not just [to reduce] emergency visits, you’ve got to look at the fact that we’ll decrease the amount of spirometry needed at the hospitals, the full pulmonary function if they only want spirometry. The [RTs] that are freed up - Freed up to deal with seeing sick patients.” – Participant 6, Interview

Participants valued the increased access for their patients to COPD-specific care, within a primary care setting.

“A significant barrier to healthcare is access – FHT/FHO/family physician offices are generally more accessible (local) than hospitals or specialized clinics. [The] BCC program benefits patients by offering easier access to another HCP [Health Care Provider] and tools previously unavailable.” – Participant 4, Living Document 1

When asked about their motivation to implement BCC, participants described a need for increased support for patients and providers regarding COPD care. BCC provided patients with more time to both discuss and learn about their disease and treatment options. Providers felt this time was valuable for both themselves and their patients.

“In my opinion, patients are looking for time with HCP’s to explain their concerns and receive education /feedback etc. Time is a luxury in healthcare, and I feel we do offer a lot of time and education to every patient.” – Participant 5, Living Document 1

The quality of the program was often cited by participants as a key benefit of implementation. Participants explained that BCC standardizes the quality of care and ensures that all patients get access to the same care. Providers valued the self-management focus of BCC and described the program as an interactive and engaged relationship between providers and their patients. One participant shared that “[b]y placing a focus on the patient during every appointment. Ensuring that they understand all of the information being discussed, they have opportunity for questions, and that I look at their overall health and seek any opportunity to help” (Participant 2, Living Document 1).

The decision to implement was also influenced by the support and guidance offered by BCC leadership and the implementation team. Participants noticed the interprofessional composition of the implementation team and how it facilitated peer-to-peer learning. From the beginning, healthcare professionals heard and learned from peers (of the same profession) about the goals, challenges, and successes of the program. The leadership team (consisting of RTs, physicians, and administrators) were available throughout implementation, bolstering participants’ readiness to implement. Frontline providers (e.g., physicians and RTs) were integral to the implementation; almost all participants indicated that having an RT as a core member of the implementation team was vital to overall success. A participant discussed that the implementation team supported all clinicians, centralized the information, and ensured that the messaging (program objectives, provider roles) was consistent from the outset. Additionally, participants valued the “physician-to-physician” role and considered it to be integral to growing a common understanding and increasing commitment (and buy-in) to the BCC program.

A key task of the peer-led implementation team was support in patient recruitment. Recruitment was initially led by the BCC implementation team in collaboration with providers at the implementation sites. BCC’s recruitment strategy involved the RTs “proactively searching [the electronic medical records] for patients who would benefit from the program” (Participant 6, Living Document 1). This initiated provider empowerment as well as surfaced possible future barriers to delivery and evaluation. Participants appreciated the proactive approach to patient recruitment as opposed to waiting for referrals.

The majority of the participants stated their readiness to implement was strengthened with the knowledge of the growing evidence of positive outcomes from the BCC program in other sites. As more sites implemented the BCC program, there was a feeling of not wanting to be left behind.

### Transformative and enabling mechanisms

Three transformative mechanisms were key to supporting the successful implementation: (1) adaptive process, (2) provider empowerment, and (3) embedded evaluation. These three transformative mechanisms were buttressed by four enabling mechanisms: (1) provider training, (2) administrative support, (3) role clarity, and (4) patient outcomes.

1. Adaptive Process

An adaptive process was key in supporting implementation. While the structure of the BCC program was largely prescribed, how the program was implemented was flexible and was often adapted to different practice settings. For example, BCC implementation was adapted based on the funding model of the clinic, clinic capacity, and space.

Program delivery needed to adapt to resources such as administrative capacity and space. The administrative staff were key to supporting implementation and embedding the program in usual care. These staff were well-positioned to increase awareness of the program among patients and adapt BCC delivery to improve efficiency based on current work practices.

“even the receptionist at one of my sites, she at first – patients would come up to the window to see social work and they’d be huffing and puffing, and she didn’t really acknowledge it. But before I left there she was saying, ‘Oh my God, are you OK? Do you need to see our RT? We have a RT.” – Participant 5, Focus Group

Primary care providers acknowledged the program’s easy incorporation into the day-to-day workflow and credited the support from administration staff. In the early implementation, participants described having to spend more time with program elements. A few participants felt this occupied a considerable amount of time and was seen as a challenge to the early delivery and workflow of BCC.

One participant expressed that during early implementation, BCC activities absorbed more time than any other resource:

“Finding patients, then booking them (if they answer phone), then the initial appt. is 1.5 hours, which is completely necessary, and the consultation … chasing down doctors, waiting outside of their rooms to get approval or simply discuss appointment and finally charting which takes up quite a lot of time.” – Participant 9, Living Document 4

BCC’s implementation was an evolving process as the program was adapted by the clinic for its unique context; clarity about roles and responsibilities grew as the clinic worked through the implementation process. This adaptive feature of the program meant that the program required an upfront investment of time and resources which was key in facilitating buy-in from different stakeholders as they progressively integrated the program into their routine activities. For example, administrative staff were key in securing role clarity and trust, through methods such as reminder calls (to patients with access to telephone) to minimize last-minute cancellations and no-shows.

As the implementation process progressed, the program was able to adapt to the processes of the site and integrate within the day-to-day practice to reduce this significant time commitment.

(2) Empowerment & Collaboration

Empowerment was embodied and discussed in several ways; namely, the empowerment of staff to deliver the services associated with the program, and the subsequent empowerment of patients and caregivers to better manage their COPD. Interprofessional collaboration was identified as a key strength of the program as it was an opportunity where staff were “working with the doctor rather than against the doctor, and ideally working with respirologists” (Participant 1, Focus Group). This was a function of time (i.e., increased practice with program delivery, and understanding roles within the program) and observing positive patient outcomes. A provider shared that “there’s a lot of … collaboration that didn’t happen before this [program]… [this has] increased our ability to do our jobs better too” (Participant 2, Interview). Document analysis confirmed this collaboration as a priority, and essential in establishing a self-management plan for the patient. One participant shared that “[t]here have been some challenges such as getting all team member[s] on the same page but over time the program has built trust and has proven its worth” (Participant 6, Living Document 1). Lack of role clarity in early implementation was also described as a barrier. For example, participants felt a lack of communication with all clinic providers and staff gave rise to ambiguity about program roles and objectives; “certain health care professionals felt their toes were being stepped on by the BCC program” (Participant 3, Living Document 5). Additionally, this initial lack of role clarity was perceived as a major challenge to the development of trust and professional relationships; “I feel that a major challenge exist[s] in the understanding of “just what we do”. All HCP’s have been supportive of my presence but not always supportive of talking about the patient right away” (Participant 5, Living Document 2).

Participants believed that achieving empowerment and collaboration could have occurred sooner with more up-front provider training related to clarification of roles and purpose of the program. As the BCC program was able to integrate into the site, the roles of BCC and primary care providers evolved. Participants explained how some physicians initially lacked understanding of the RT roles within the program; one participant felt this lack of understanding may lead to physicians being reluctant to refer patients or give RTs patient information. Multiple participants felt all clinic providers and administration should be trained on the program’s offerings early in implementation. Participants noted that there was an increase in engagement at the clinician and administrative level after the program had been operating for some time and they became more familiar with it. For example, physicians started adding patients to the RT’s schedule, making patient recruitment easier. Embedding the RT on-site, co-located with the primary care provider, helped to enhance role clarity during implementation through regular contact and communication.

Participants noticed as administrative staff developed a clearer understanding of their role and the function of the program, they provided increased support through reminder calls and managing appointments. Participants also noted that in early implementation, higher level of no-show and cancellation rates (more common in patients with barriers to access as well as lack of stable housing, internet, and phone) was, in part, attributable to a lack of role clarity of support from administration and other clinicians. When administrative support was strengthened, there was better patient attendance. As the implementation process progressed and the program became aligned with the internal processes of the site, the program as able to utilize the support from staff to ease the delivery of the BCC program.

(3) Embedded Evaluation

Evaluation made providers aware of the value of the educational component of the program; “they’re understanding their disease, they’re understating why they’re in seeing us. And at first they’re hesitant sometimes at an hour-and-a-half appointment, but I’ve never had anybody upset that they came” (Participant 3, Focus Group). One participant noted that they typically see the benefits of the program within a year. Another participant noted that the impact of the program is demonstrated in decreased patient’s COPD Assessment Test (CAT) scores. The CAT score is a validated measure of disease-specific quality of life.

Embedded evaluation meant patient outcomes were constantly and consistently reported. For most participants, the regular appointments allowed for both formal and informal evaluation. Participants were able to see, first-hand, positive improvements. One participant shared that they “measure [patient outcomes] from comparing their knowledge starting the program compared to today. The patients review their action plans and device technique at most follow ups which demonstrates knowledge and understanding of our program” (Participant 9, Living Document 4).

This was coupled with patients’ positive responses to their appointments. Participants described patients as being receptive to the education, recalling for example patients saying “nobody’s ever shown me this, nobody’s ever explained this to me” (Participant 3, Focus Group). Participants also believed that the program empowered patients by improving patients’ self-efficacy by equipping them with the skills, knowledge, and confidence to manage their COPD. One participant noted that BCC “give[s] patients the power and knowledge to understand their disease, symptoms, and management so they can take control of their own health” (Participant 9, Living Document 1).

There were metrics available such as patients’ CAT and the Modified Medical Research Council (mMRC) breathlessness scores (taken at every appointment), and healthcare services utilization data such as hospital admissions, emergency department visits, and consultations with physicians. One participant expressed that “people like data … even if they don’t truly understand it” (Participant 5, Living Document 6). As the program was implemented across the region, there was an increase in the quantity and diversity of the data available, which, in turn, solidified the program in existing sites and further facilitated progressive implementation to new sites.

## Discussion

**Fig. 1:**
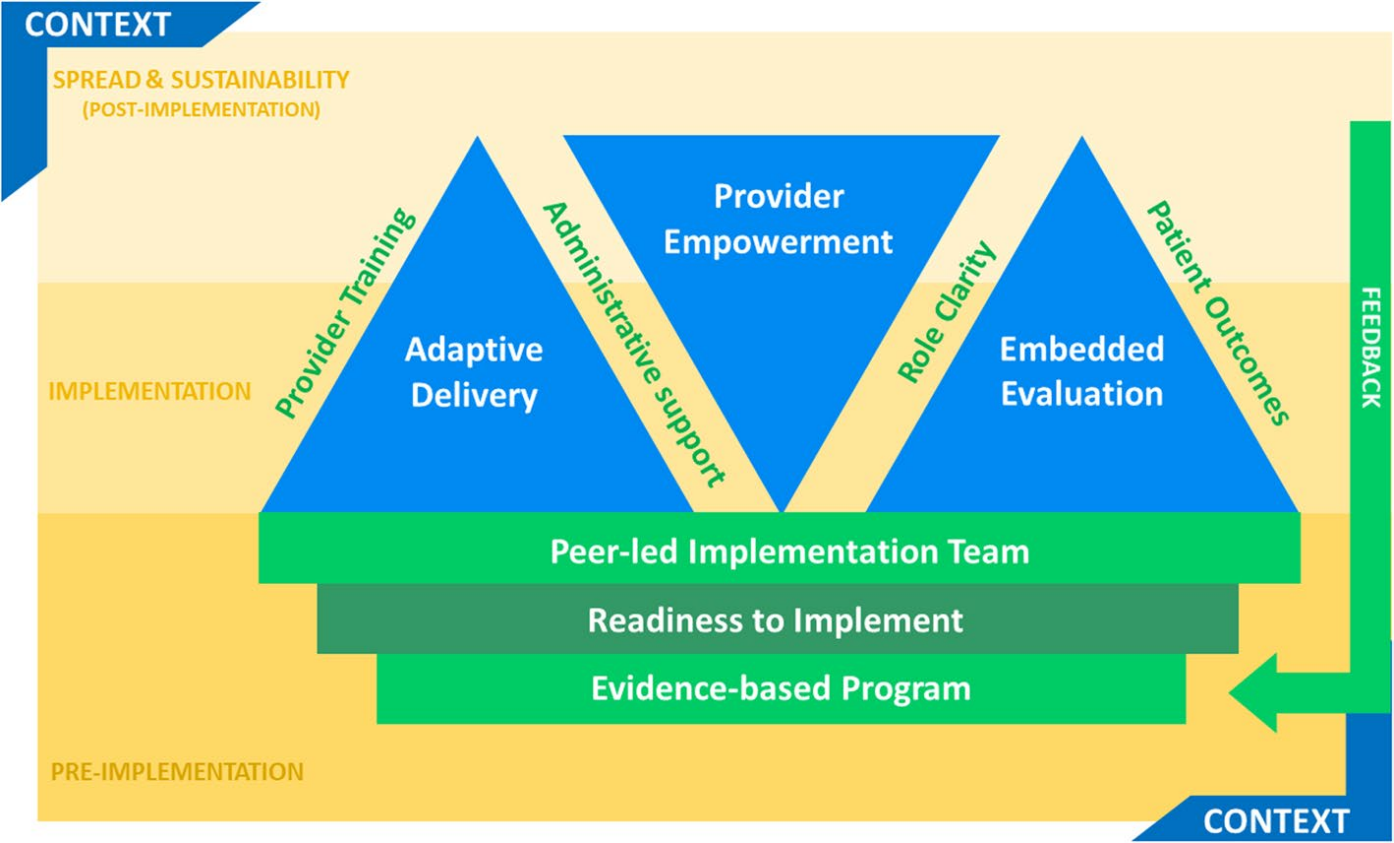
Implementation phases and mechanisms

BCC is understood to be a high-quality program with demonstrated improved patient outcomes and increased provider satisfaction. BCC was implemented in a primary care setting which is a reliable point of intervention for chronic disease management programs, and more specifically, COPD management programs [29]. The program has improved patients’ ability to access the appropriate care in the appropriate setting [30]. By equipping patients with the knowledge and skills to manage their COPD, BCC improved health literacy [31] by empowering patients to be proactive partners in their own care. This approach is increasingly being viewed as a promising solution to address the complex needs of patients with chronic disease as it allows for the creation of care plans informed by patients for patients [8]. Successful implementation of an integrated team-based care model is a complex and multi-faceted process. Our research explored the progressive implementation of the BCC program and in doing so exposed some of this complexity. We propose a framework to support progressive implementation that is framed by context; it contains three phases and 10 mechanisms observing the interplay between the mechanisms across the three phases of implementation including pre-implementation, implementation, and spread and sustainability (i.e., post-implementation) (Table 1; Fig. 1).

Enabling mechanisms (provider training, administrative support, role clarity, and patient outcomes) worked collectively across the transformative mechanisms (adaptive process, provider empowerment, embedded evaluation). Our results suggest that implementation strategies must deviate from the traditional linear approach [16, 32]. Instead, successful implementation must encompass interconnected and symbiotic mechanisms that consider the dynamic nature of the system and adapt to unpredictability and uncertainty within the unique context [16, 32]. We found that all the mechanisms were at play across all sites in our study and were difficult to tease apart, however, some mechanisms required varying degrees of effort as sites progressed through implementation. Significant effort and time were needed early in implementation to ensure adaptive delivery and embedded evaluation; too often a lack of embedded evaluation can result in inappropriate delivery methods which, in turn, give rise to inconsistent outcomes [33]. The opposite was true for provider empowerment, where providers reported feeling more empowered as their confidence in the program, its delivery, and outcomes increased; implementation could be described as an ‘inside-out’ approach where sites were the source and destination for a change in care delivery [34].

Post-implementation refers to the time when providers start to focus more on sustaining the program within the current site and program leaders focus on spreading into new clinics. The foundational, transformative, and enabling mechanisms at play during implementation remain active in post-implementation, although with less effort required. Demonstrated outcomes and word-of-mouth work to increase awareness of the program in other sites which, in turn, contributes to positive staff morale and staff buy-in when implementing in other sites [35, 36]. Altogether, there is an improvement in the ease of implementation [8, 29].

Adaptive delivery and embedded evaluation both require a high investment of time and resources during the initial stages of implementation when the program is unfamiliar to staff and patients. A flexible approach to implementation has been shown to improve the likelihood of success in implementation [37]. In our study, once implementation was complete and the program was in full delivery, providers felt more empowered. Early efforts of adaptive delivery and embedded evaluation could be waned, as they became part of regular care. Clinic buy-in peaked in post-implementation as staff assumed day-to-day support of BCC; concurrently, the program’s workflow processes gradually integrated with the clinic activities and clinic staff took on more of the day-to-day support for the program.

During implementation, cancellations and duration of the appointments presented challenges with some sites for program delivery. Participants felt this was especially relevant when working with patients who experience barriers in access to care due to a lack of stable housing, telephone, and/or internet [38]. Despite these challenges, BCC’s proactive recruitment strategy (i.e., finding patients who would benefit from the program, as opposed to waiting for referrals) was a key strategy to successful implementation. This was crucial during the initial stage of implementation and helped to expose challenges for implementation and delivery.

Embedding evaluation required a significant amount of work during initial implementation however, this effort waned as implementation progressed. Collecting and sharing data from implementation sites is key not only in sustaining program success, but also in laying the foundation for future implementation success [39]. In this study, this knowledge of improved patient outcomes and provider satisfaction was shared by word-of-mouth and through the peer-to-peer implementation team. These strategies, along with more traditional academic dissemination strategies, supported progressive implementation.

With the BCC program, providers were able to enhance a patients’ self-management and improve access to appropriate care, resulting in overall improved patient care and improved provider satisfaction. This was accomplished with relative ease; participants were supported at each phase of implementation by a peer-led implementation team and continued support was maintained through peer-to-peer learning. Research shows that a program is more likely to be successfully implemented when there is adequate support coupled with relative ease of implementation [40, 41].

Participants unanimously agreed that the BCC program was effective in improving the self-efficacy of the patients by supporting the development of their knowledge, skills and ultimately, confidence to manage their condition, and these findings are consistent with the literature [42–45]. Interventions that improve self-efficacy have demonstrated success in improving health outcomes, compared to traditional patient education strategies which give patients information about their conditions but fail to give them the skills or confidence to apply this information [46, 47].

Role clarity also supported implementation. Clearly defined roles and each team member’s contribution to COPD care are essential to facilitating the collaboration needed for implementation [9]. This may be an indication that program implementation is especially efficient when implemented in a team that already offers interprofessional care and one that is well integrated with the organization’s structure [48]. It follows that the implementation of a program into a high-functioning team will require less overall effort [49]. Having the educators co-located with the primary care providers enhanced the communication and collaboration among the providers, promoting the exchange of knowledge to facilitate the implementation process [50, 51]. Interprofessional teams that are able to work together in one location have been seen to optimize role clarification and support integrated health services [52]. Furthermore, the function of provider empowerment evolved during the course of implementation; as providers became more aware of their roles, their empowerment enabled increased patient recruitment and ease of program delivery coupled with integration within the existing workflow of the clinic [53, 54].

Support from the administrative staff was a key resource in overcoming implementation barriers. In addition to facilitating communication (provider-patient and provider-provider), administrative staff understood the flow of resources – notably space, time, and personnel. As administrative staff became more aware of the program, its objectives, and their role within it, this allowed for efficient implementation, program delivery, and integrated workflow.

## Limitations

Qualitative research poses unique challenges to the generalizability of findings and this study is no exception. The aim of this study is to share lessons from one example of progressive implementation as opposed to providing overarching recommendations. Accordingly, we believe the lessons learned are transferable to other settings and contexts.

The primary limitation of this study relates to sample size and response rates, and we used a rigorous approach to our case study (multiple methods across multiple sites) to mitigate this limitation. More specifically, we acknowledge that our small sample size and variability in response rates may allow for potential biases to impact the data. For example, the study did not include results from patients and their caregivers, and this can pose the possibility of bias, especially in findings that report success based on perceived patient-centered outcomes. We would like to highlight that these groups were included in our larger research program which may serve to limit the influence of potential bias and its impact on the results.

Among the participants that were included in this study, especially in the focus group, there is a potential for controversial or unpopular views to be suppressed which can give rise to false consensus [55]. Inclusion of a variety of data collection tools such as LDs and document analysis provided staff with an opportunity to share their individual insights. The data collected through these tools were consistent with the data collected through the focus group, suggesting that the focus group findings were representative of participants’ views. It is important to note that this study also involved member checking to provide another opportunity for the research team to ensure that their analysis of the data was accurate [27, 28]. Furthermore, there also may be an increased likelihood of the suppression of negative opinions if participants are direct providers of the BCC program or considered to be ‘insiders’ [56]; we mitigated the ‘insider effect’ by including the perspective of care providers who, prior to implementation, were external to the BCC program.

Finally, it would be remiss to not mention the impact of the COVID-19 pandemic on this work, however, it is difficult to fully assess its influence. Our data collection was concluding at the beginning of 2020, as the effects of the pandemic were beginning to impact the region. As a result, our study did not explore, nor do we believe it was impacted by the pandemic. The pandemic has shifted how healthcare operates; this may change the focus from co-location to collaboration in regards to integrated team-based care [52]. Further research is needed to understand how the pandemic has impacted the progressive implementation of BCC and other integrated, team-based models for chronic disease management.

## Conclusion

The rapidly increasing prevalence of chronic diseases, and COPD more specifically [57], emphasizes the need to better support patients and providers in the implementation of appropriate models of care [5]. The successful implementation of the BCC program led to improved management of COPD, quality of patient care, and patient and provider experience. This case study explored mechanisms that support the progressive implementation of integrated team-based care within the context of COPD. While BCC has been applied within the context of COPD, the insights gained from this study can inform the application of the program in the context of other chronic diseases. The performance of BCC at various sites in Ontario suggests that integrated team-based care has the potential to manage the growing impact of chronic disease on Canadians and subsequent burdens on the healthcare system.

## Data Availability

The datasets generated and/or analysed during the current study are not publicly available due to privacy and confidentiality but are available from the corresponding author on reasonable request.

## Abbreviations

ARGI: Asthma Research Group Inc.
BCC: Best Care COPD
CFIR: Consolidated Framework for Implementation Research
COPD: Chronic obstructive pulmonary disease
EPIS: Exploration, Preparation, Implementation, and Sustainment
PARIHS: Promoting Action of Research Implementation in Health Services
RT: Respiratory therapist
SRQR: Standards for Reporting Qualitative Research
